# Training the Social-Emotional Skills of Youth School Students in Physical Education Classes

**DOI:** 10.3389/fpsyg.2021.741195

**Published:** 2021-09-08

**Authors:** Romualdas Malinauskas, Vilija Malinauskiene

**Affiliations:** Department of Social and Physical Education, Lithuanian Sports University, Kaunas, Lithuania

**Keywords:** training program, youth schools, students, physical education classes, social-emotional skills

## Abstract

This study aimed to examine the impact of implementing an innovative Social-Emotional Skills Training Program in physical education classes at youth schools. This study used two surveys: the Schutte Self-Report Inventory (otherwise known as the Emotional Intelligence Scale) and the Social Skills Rating System (student form). The analysis included 104 youth school students (Mage = 16.91; SD = 1.12), who were randomly selected from two youth schools in the Kaunas region. Four classes were randomly assigned into an experimental (*n* = 49) and a control (*n* = 55) group. The experimental group participated in the Social-Emotional Skills Training Program. The intervention was targeted at the following social-emotional skills: empathy, cooperation, assertion, self-control, optimism, ability to understand and analyze emotions, appraisal, and utilization of emotions. The modified physical education classes were conducted by the physical education teacher, who was instructed by the researcher. Repeated measures multivariate ANOVA was used to analyze the effects of the Social-Emotional Skills Training Program. During the experiment, the applied training procedures had a statistically significant effect on the social-emotional skills of the experimental group of youth school students. Thus, the findings demonstrate that this program (for enhancing social-emotional skills of youth school students) positively impacted the social-emotional skills of the students. These results highlight the need to consider social-emotional skills training factors when interpreting the level of social-emotional skills among youth school students.

## Introduction

Recent scientific literature broadly supports the notion that social-emotional skills are important for personality development, the development of prosocial behavior, and positive emotional growth. Social-emotional skills have been described as a multidimensional construct that includes the abilities to manage emotions, feelings, and care and concern for others, as well as to solve problems and have positive peer relationships (Zins et al., 2004). Various reviews and meta-analyses have proven the effectiveness of implementing social-emotional skills training in the general education context (Durlak et al., 2011; Korpershoek et al., 2016; Taylor et al., 2017; Corcoran et al., 2018). However, only a few reviews have analyzed the effectiveness of social-emotional skills training in the sports or physical education context (Rasberry et al., 2011; Bessa et al., 2019, 2021). Meanwhile, a systematic review revealed that only 19 studies (37%) confirmed the fidelity of implementing the educational program, meaning “the authors performed the validation of the model implementation and presented a detailed description of the program.” (Bessa et al., 2019, p. 824)

Youth schools have been chosen for this study because they are designed to teach students who have dropped out of typical schools and characteristically lack strong social-emotional skills (Malinauskas, 2019). Although youth schools are “usually part of the middle or high school program offered to secondary-aged students” (Dunning-Lozano, 2016, p. 434), youth schools in Lithuania are the alternative schools established to meet the behavioral and educational needs of adolescents and adults that cannot be properly met in a traditional school setting. Many students in youth schools have substantial academic and behavioral problems (Foley and Pang, 2006), such as attendance problems, underachievement problems, and having insufficient credits to graduate (Dunning-Lozano, 2016). Many students at youth schools have been sent there to prevent them from interfering with other students after they were repeatedly suspended due to fighting or disrupting classes. Youth school students can have unique learning interests and learning barriers, and they can be potential perpetrators of crimes or misdemeanors, even participating in the juvenile detention system (Malinauskas and Saulius, 2019).

Training the social-emotional skills of youth school students in physical education classes is a viable proposition because physical education classes promote intense emotions and can foster a positive environment that promotes social-emotional learning (Gagnon, 2016; Escartí et al., 2018). Physical education can be considered a school subject where students and teachers can create positive experiences (Dyson et al., 2021). Notably, previous studies have confirmed the relationship between physical activity/education and improved social-emotional indicators (Fernandez-Rio and Menendez-Santurio, 2017; Cañabate et al., 2018). These studies have indicated improved social responsibility, cooperation, solidarity, self-control, and self-esteem, among other factors. Research evidence suggests that quality physical education contributes positively to the development of social-emotional skills of the students (Hunter, 2006) and that physical education classes develop social-emotional skills in the affective domain, for example, in the context of “controlling one's emotions during competitive game play…and demonstrating awareness of and support for other classmates' differences.” (Ciotto and Gagnon, 2018, p. 28) With better-developed social-emotional skills, students are more likely to seek help when needed, can better control their emotions, and can solve problems more successfully in different situations (Romasz et al., 2004). Acquiring social-emotional skills provides an opportunity to be successful not only during physical education classes but also in the context of everyday life (Ciotto and Gagnon, 2018).

Given that implementing educational programs must be theoretically grounded, we chose the model-based practice of cooperative learning (Dyson and Casey, 2012) for this study because model-based practices have been posited as fundamental tools for helping students accomplish social-emotional learning outcomes (Jacobs and Wright, 2014). The theoretical cooperative learning model integrates the study variables and is effective for developing social-emotional and relationship skills “amongst mainstream students and students with moderate/special educational needs amongst elementary and middle school ages.” (Dyson et al., 2021, p. 9).

There is a considerable need for in-depth research aimed at better understanding the particularities of training the social-emotional skills of youth school students in physical education classes. Youth school students have been exposed to different types of trauma, producing individual discrepancies in the need for social-emotional skills training. Better understanding the social-emotional skills of youth school students through physical education classes could help them transform their weaknesses into strengths and enable them to overcome the challenges they face.

To examine the effects of this educational program on the social-emotional skills of youth school students, we investigated the changes in the social-emotional skills of the youth school students following the implementation of the educational program in physical education classes. Given that previous studies have proven the effectiveness of training the social-emotional skills of secondary school students during physical education classes (Sklad et al., 2012; Akelaitis and Malinauskas, 2016; Escartí et al., 2018; Bartlett, 2019), we hypothesized that youth school students would demonstrate significantly improved social-emotional skills following the implementation of the program.

## Methods

### Context and Participants

A random serial sampling method was used for the educational experiment: first, two youth schools were selected from the list of district schools; then, four classes were selected from the class lists of the selected schools. The analysis included 104 youth school students (Mage = 16.91; SD = 1.12), who were randomly selected from two youth schools in the Kaunas district. Four classes were randomly assigned into an experimental (*n* = 49) and a control (*n* = 55) group. The inclusion criteria for study participants were middle adolescence (15–18 years) and male or female, and the only exclusion criterion was refusing to give informed consent. The research used a two-group pretest and posttest study design. The experiment was conducted during the 2019–2020 academic year. There were no significant differences between the experimental (16.71 ± 1.17) and control (17.09 ± 1.06) groups in terms of age (*t* (102) = −1.72; *p* > 0.05) or gender (experimental group: 37 boys and 12 girls; control group: 36 boys and 19 girls) [χ^2^ (1) = 1.25; *p* > 0.05 (*p* = 0.26)].

### Instruments

We used two instruments to measure the social-emotional skills of youth school students. Emotional skills were measured using the Schutte Self-Report Inventory (SSRI), which was validated by Schutte et al. (1998). The SSRI, also known as the Emotional Intelligence Scale, the Self-Report Emotional Intelligence, and the Schutte Emotional Intelligence Scale, assesses emotional intelligence skills based on self-reported responses. This scale was designed to determine participant perceptions of their emotional skills at both intrapersonal and interpersonal levels. It comprises 33 items answered on a 5-point Likert scale (where 1 = strongly disagree, 2 = disagree, 3 = neutral, 4 = agree, and 5 = strongly agree) such as “I like to share my emotions with others,” “I expect that I will do well on most things I try.” Higher values represent a higher emotional skill level. Various authors have considered psychometric properties and concluded that this scale is useful as a brief measure of emotional intelligence (Petrides and Furnham, 2001). The instrument usefully divides emotional intelligence into four separate skills (Palmer, 2003): the ability to use his/her own positive emotional experiences (optimism), the ability to assess and express emotions (appraisal), the ability to understand and analyze emotions (emotional understanding), and the ability to manage emotions (utilization). The research demonstrated good internal consistency (α = 0.81). The Lithuanian version of the SSRI demonstrated an internal consistency value of 0.79 for the overall questionnaire (Malinauskas and Sniras, 2010).

Meanwhile, the Social Skills Rating System (Student form) (SSRS-S; Gresham and Elliott, 1990) comprises a self-reported questionnaire for students in grades 7–12, which comprises 39 statements requiring each student to respond with how frequently they demonstrate the behavior described and their perception of the importance of that behavior, for instance, “I make friends easily (assertiveness)” and “I follow the teacher's directions (cooperation).” The assessment method was based on the four dimensions (skills) defined by Gresham and Elliott (1990): cooperation, assertiveness, empathy, and self-control. Each item was rated on a 3-point frequency scale (0 = never, 1 = sometimes, and 2 = many times). This study found a Cronbach's alpha of 0.63 for the total SSRS-S score. The Lithuanian version of the SSRS-S ranges from 0.66 to 0.76 (Griciute et al., 2008).

### Intervention (Educational Program)

To verify the efficiency of the educational program, we used an educational experiment method. The experimental group participated in a Social-Emotional Skills Training Program that included 48 15-min sessions (total of 12 h) during modified physical education classes conducted before the COVID-19 pandemic (i.e., September 2019 until March 2020). We targeted the intervention at the following social-emotional skills: empathy, cooperation, assertion, self-control, optimism, ability to understand and analyze emotions, appraisal, and utilization of emotions. The same amount of development time was allocated to each social-emotional skill (90 min). All of the youth school students in the experimental group attended 15-min training sessions four times per month during the modified physical education classes. The control group did not partake in any training sessions. The modified physical education classes were conducted by the physical education teacher, who received instructions from the researcher. To implement the social-emotional skills training during physical education classes, five training stages were incorporated as follows: (1) skill description; (2) skill demonstration; (3) skill practice; (4) feedback; and (5) reinforcement of trained skill. Table 1 provides details of the Social-Emotional Skills Training Program for youth school students in physical education classes.

**Table 1:** Description of social-emotional skills training program for youth school students in physical education classes.

**Intervention**	**Number of sessions**	**Content**	**Goals**	**Training methods**
**Emotional skills**
	6	Utilization	Learn and practice to manage emotions	Method of impulse control (autogenic training), discussion, and group learning (formally termed cooperative learning)
	6	Appraisal	Learn and practice to assess and express emotions	Role play scenarios, discussion, and group learning
	6	Emotions' understanding	Learn and practice to understand and analyze emotions	Watching the video, discussion, group learning, and written worksheets
	6	Optimism	Learn and practice to use own positive emotional experience	Role play scenarios, discussion, group learning, and written worksheets
**Social skills**
	6	Empathy	Learn and practice to understand thoughts and feelings of other students	Watching the video about youth school student in physical education classes, discussion, group learning, and written worksheets
	6	Cooperation	Learn and practice social skills, such as cooperation and compromise	Role play scenarios, discussion, and group learning
	6	Assertion	Learn and practice to communicate in an open and positive way without being either aggressive, or passively accepting “wrong”	Role play scenarios, discussion, group learning, and written worksheets
	6	Self-control	Learn and practice to demonstrate self-control and respect for others	Method of impulse control, discussion, and group learning

The training methods applied by the Social-Emotional Skills Training Program included impulse control (autogenic training), post-activity discussion about the shared experiences, group learning (cooperative learning), role-play scenarios, watching the videos, and written worksheets of the students. For example, when the youth school students had to improve their emotional management skills, autogenic training methods were adopted, including relaxation and impulse control methods. When working on understanding and analyzing emotions, students watched real-life videos demonstrating the difficulties suffered by students during the communication process. Following the video demonstration, the youth school students had to answer questions (e.g., “If your friend is sad, and you would like to say something to your friend about their mood or feeling during physical education classes, what would you say?”). Then, students were grouped into pairs to practice emotional understanding better (group learning). Later, the students jointly considered their emotional experiences (discussion about their shared experiences). The analogous methods were utilized during each session.

After 3 months, students from the experimental group had accomplished the interventional aims of the educational program and had improved all of their social-emotional skills. The students from the experimental group wrote in a free-answer form that they had improved empathy, cooperation, assertion, self-control, optimism, the ability to understand and analyze emotions, appraisal, and utilization of emotions. Participants from the experimental group also assessed the Social-Emotional Skills Training Program in the context of this free-answer form, reporting that they were satisfied with the educational program and had improved their relationships with their classmates.

### Data Collection

Before the data collection, ethical clearance was obtained from the Ethical Committee of the Lithuanian Sports University. The study was conducted in accordance with the Declaration of Helsinki. Parental consent was obtained prior to the experiment because the participants were aged between 15 and 18 years. Confidentiality was ensured because the questionnaires were distributed for completion in classrooms, and participant names were not recorded.

The data collection procedure was realized by providing students with sufficient time to answer the questions in the classroom. The physical education teacher administered the questionnaires in the classroom. The time participants took to complete the SSRI varied between 12 and 15 min. The time participants took to complete the SSRS-S varied between 15 and 18 min. The study was conducted in three stages: first, the pre-assessment of the variables for the experimental and control groups; second, the implementation of the educational program for the experimental group (with the control group continuing with their traditional training); and third, following the implementation of the intervention, the posttest assessment of variables was conducted for both groups.

### Data Analysis

The experimental design of this study included a quantitative analysis of the data from the experimental (intervention) group and the control (comparison) group. We calculated means (M) and SDs for each variable. Skewness and kurtosis coefficients were calculated to verify the assumption of the data normality using multivariate ANOVA (MANOVA). In general, skewness and kurtosis values between −1 and 1 reflect the data normality. Pearson's correlations (two-tailed) were calculated for all variables. A 2 (Group: experimental group and control group) × 2 (Time: before the experiment and after the experiment) repeated measures (RM) MANOVA followed by the one-way ANOVA was used to investigate differences between Group and Time interactions in terms of social-emotional skills. Preliminary assumption testing checked for multicollinearity, sphericity, homogeneity, and equality of variance/covariance matrices; no serious violations were noted.

Wilks' Lambda statistic was used to evaluate the significance of multivariate effects, and the alpha level was set to 0.05. The effect sizes for *F*-statistics were expressed as partial eta-squared ($\eta_{\text{p}}^{2}$). Statistical analyses were processed using SPSS software (version 21.0).

## Results

Table 2 illustrates the computed statistical notations (means, SDs, skewness and kurtosis, and SEs of skewness and kurtosis) for all examined variables. All measures were checked for skewness and kurtosis and were considered to have acceptable distributions because they were between −1 and 1. This indicated that the assumptions for the data normality were met and confirmed the viability of using the Student's *t*-test and the RM-MANOVA.

**Table 2:** Means (M), SD, and normality tests of the study variables (*N* = 104).

	**Before experiment**						**After experiment**					
	**M**	**SD**	**Sk**	**SkSE**	**Ku**	**KuSE**	**M**	**SD**	**Sk**	**SkSE**	**Ku**	**KuSE**
Utilization	3.23	0.52	0.40	0.24	0.15	0.47	3.60	0.56	0.12	0.24	0.01	0.47
Appraisal	3.37	0.35	0.26	0.24	−0.57	0.47	3.68	0.35	−0.10	0.24	0.09	0.47
Emotions' understanding	3.33	0.43	0.99	0.24	0.88	0.47	3.57	0.43	0.59	0.24	0.22	0.47
Optimism	3.34	0.28	0.28	0.24	0.15	0.47	3.78	0.35	0.15	0.24	0.27	0.47
Total emotional skills	3.24	0.26	0.40	0.24	−0.31	0.47	3.69	0.32	0.73	0.24	0.59	0.47
Empathy	12.40	1.19	−0.48	0.24	0.95	0.47	14.83	1.82	0.45	0.24	0.05	0.47
Cooperation	11.98	0.52	−0.03	0.24	0.81	0.47	14.85	1.72	−0.01	0.24	−0.88	0.47
Assertion	11.65	1.03	0.31	0.24	−0.31	0.47	13.64	1.90	−0.41	0.24	0.69	0.47
Self-control	12.68	1.05	−0.14	0.24	0.39	0.47	14.58	1.65	0.25	0.24	−0.48	0.47
Total social skills	48.71	2.66	0.28	0.24	0.32	0.47	57.88	5.13	0.34	0.24	0.21	0.47

*Sk, Skewness; SkSE, Skewness SE; Ku, Kurtosis; KuSE, Kurtosis SE*.

Pearson's bivariate correlation coefficients were calculated to investigate the relationships between all dependent variables and the absence of multicollinearity. Given that no correlation exceeded 0.85, the assumption of multicollinearity was not violated (Weston and Gore, 2006). Pearson's bivariate correlation coefficients between variables are presented separately for the Time before the experiment and the Time after the experiment in Table 3. As shown in Table 3, the social-emotional skills of the students were not very substantially correlated in the positive direction.

**Table 3:** Correlations of dependent variables.

	**1**	**2**	**3**	**4**	**5**	**6**	**7**	**8**	**9**	**10**
Utilization	1	0.35^**^	0.53^**^	0.46^**^	0.69^**^	0.32^**^	0.22^*^	0.20^*^	0.22^*^	0.33^**^
Appraisal	0.29^**^	1	0.45^**^	0.45^**^	0.64^**^	0.42^**^	0.39^**^	0.45^**^	0.40^**^	0.58^**^
Emotions' understanding	0.36^**^	0.39^**^	1	0.54^**^	0.82^**^	0.43^**^	0.38^**^	0.32^**^	0.28^**^	0.48^**^
Optimism	0.42^**^	0.27^**^	0.22^*^	1	0.85^**^	0.35^**^	0.40^**^	0.34^**^	0.39^**^	0.50^**^
Total emotional skills	0.68^**^	0.61^**^	0.71^**^	0.71^**^	1	0.48^**^	0.47^**^	0.39^**^	0.42^**^	0.60^**^
Empathy	0.40^**^	0.40^**^	0.20^*^	0.43^**^	0.49^**^	1	0.43^**^	0.48^**^	0.32^**^	0.78^**^
Cooperation	0.39^**^	0.24^*^	0.27^**^	0.29^**^	0.43^**^	0.20^*^	1	0.32^**^	0.24^*^	0.68^**^
Assertion	0.19^*^	0.42^**^	0.37^**^	0.39^**^	0.47^**^	0.34^**^	0.33^**^	1	0.39^**^	0.77^**^
Self-control	0.27^**^	0.33^**^	0.22^*^	0.25^*^	0.33^**^	0.30^**^	0.27^**^	0.35^**^	1	0.66^**^
Total social skills	0.44^**^	0.51^**^	0.38^**^	0.50^**^	0.62^**^	0.74^**^	0.52^**^	0.74^**^	0.72^**^	1

*Correlations below the diagonal are for Time before the experiment*.

*Correlations above the diagonal are for Time after experiment. N = 104*.

* *p < 0.05;*

** *p < 0.01*.

An important assumption for the univariate RM-MANOVA procedure is that of sphericity. The condition for Mauchly's test of sphericity was met because RM variables only feature two levels.

Using the Student's *t*-test for independent samples, we found that, before the experiment, the experimental and the control group did not differ significantly with respect to any of the social-emotional skills under consideration: utilization (*t*_(102)_ = 1.16; *p* = 0.25), appraisal (*t*_(102)_ = 1.58; *p* = 0.12), emotional understanding (*t*_(102)_ = 0.80; *p* = 0.43), optimism (*t*_(102)_ = 0.22; *p* = 0.83), total emotional skills (*t*_(102)_ = 1.54; *p* = 0.13), empathy (*t*_(102)_ = 1.87; *p* = 0.06), cooperation (*t*_(102)_ = 1.89; *p* = 0.06), assertion (*t*_(102)_ = 1.92; *p* = 0.06), self-control (*t*_(102)_ = −0.83; *p* = 0.41), and total social skills (*t*_(102)_ = 1.57; *p* = 0.12).

Meanwhile, the RM-MANOVA revealed significant effects of the Social-Emotional Skills Training Program on the social-emotional skills of youth school students, i.e., the influence of Group by Time interaction was significant (Wilks' Lambda = 0.67; *F*_(10, 93)_ = 4.51; *p* = 0.00). The effect was large according to Cohen ($\eta_{\text{p}}^{2}$ = 0.33), with 33% of variance accounted for by this interaction effect. According to the study by Dimitrov and Rumrill (2003), “a significant Group by Time interaction indicated that the change from pre-test to post-testing was different depending upon the treatment groups” (2003, p. 160). In plain terms, while no difference was observed between the control and experimental groups for the social-emotional skills pretest, there was a considerably sized divergence observable posttest.

Elsewhere, as shown in Table 4, the univariate RM-MANOVA testing proved the significant influence of the Social-Emotional Skills Training Program on the social-emotional skills, with effect sizes ranging from small to large. Table 4 shows that, following the intervention, students from the experimental groups demonstrated better social-emotional skills in physical education classes than the control group students, reporting higher scores for utilization (*p* = 0.000), appraisal (*p* = 0.045), emotional understanding (*p* = 0.019), optimism (*p* = 0.025), total emotional skills (*p* = 0.006), empathy (*p* = 0.004), cooperation (*p* = 0.008), assertion (*p* = 0.002), self-control (*p* = 0.034), and total social skills (*p* = 0.000).

**Table 4:** Mean scores of social-emotional skills of youth school students in physical education classes before and after the educational experiment.

	**Experimental group M (SD)**		**Control group M (SD)**		**Univariate tests of RM MANOVA** **Group** **×** **Time**		
**Social-emotional skills**	**Before experiment**	**After experiment**	**Before experiment**	**After experiment**	***F***_**1, 102**_	***p***	$\eta_{\text{p}}^{2}$
Utilization	3.29 (0.49)	3.79 (0.48)	3.17 (0.55)	3.44 (0.58)	14.50^**^	0.000	0.124
Appraisal	3.42 (0.36)	3.79 (0.31)	3.32 (0.33)	3.58 (0.35)	4.11^*^	0.045	0.039
Emotions' understanding	3.37 (0.37)	3.66 (0.37)	3.30 (0.47)	3.48 (0.47)	5.64^*^	0.019	0.052
Optimism	3.35 (0.26)	3.87 (0.31)	3.34 (0.30)	3.70 (0.37)	5.15^*^	0.025	0.048
Total emotional skills	3.28 (0.23)	3.79 (0.28)	3.20 (0.27)	3.60 (0.32)	7.98^**^	0.006	0.073
Empathy	12.63 (1.36)	15.61 (1.75)	12.20 (0.99)	14.13 (1.59)	8.44^**^	0.004	0.076
Cooperation	12.08 (0.49)	15.41 (1.72)	11.89 (0.53)	14.35 (1.58)	7.22^**^	0.008	0.066
Assertion	11.86 (1.06)	14.49 (1.67)	11.47 (0.98)	12.89 (1.78)	10.46^**^	0.002	0.093
Self-control	12.59 (0.93)	14.90 (1.62)	12.76 (1.15)	14.29 (1.63)	4.61^*^	0.034	0.043
Total social skills	49.14 (2.72)	60.39 (4.70)	48.33 (2.57)	55.65 (4.45)	19.85^**^	0.000	0.163

*$\eta_{\text{p}}^{2}$partial eta-squared;*,

* *p < 0.05;*

** *p < 0.01*.

## Discussion

The findings of the educational experiment confirm our hypothesis that the social-emotional skills of youth school students would improve significantly following the educational program based on the observation of more developed social-emotional skills in physical education classes among students from the experimental group. Following the educational experiment, the experimental group students demonstrated better utilization (a medium effect: $\eta_{\text{p}}^{2}$ = 0.124), appraisal (a small effect: $\eta_{\text{p}}^{2}$ = 0.039), emotional understanding (a small effect: $\eta_{\text{p}}^{2}$ = 0.052), optimism (a small effect: $\eta_{\text{p}}^{2}$ = 0.048), total emotional skills (a small effect: $\eta_{\text{p}}^{2}$ = 0.073), empathy (a small effect: $\eta_{\text{p}}^{2}$ = 0.076), cooperation (a small effect: $\eta_{\text{p}}^{2}$ = 0.066), assertion (a medium effect: $\eta_{\text{p}}^{2}$ = 0.093), self-control (a small effect: $\eta_{\text{p}}^{2}$ = 0.043), and total social skills (a medium effect: $\eta_{\text{p}}^{2}$ = 0.163). All of these significant changes indicate the impact of the Social-Emotional Skills Training Program on social-emotional skills as expressed in physical education classes. These findings coincide with those of other studies, such as the studies by Durlak et al. (2015), Dusenbury and Weissberg (2017), and Taylor et al. (2017), whose investigations on the effectiveness of social-emotional skills interventions produced small effect sizes (ranging from Hedge's *g* = 0.13 to Hedge's *g* = 0.23). In addition, a meta-analysis by Durlak et al. (2011) established that schoolchildren who participated in Social Emotional Skills Training Programs demonstrated significantly enhanced social and emotional skills (a small effect: Hedge's *g* = 0.26), attitudes toward school (a small effect: Hedge's *g* = 0.11), positive social behavior (a small effect: Hedge's *g* = 0.17), and academic performance (a small effect: Hedge's *g* = 0.32).

The results of this study are also consistent with various studies that have identified the significant effect of the educational programs on improvements to social-emotional skills or social self-efficacy. For instance, Malinauskas et al. (2018) evaluated the effectiveness of educational programs for enhancing social self-efficacy among basketball-playing students. The duration of the educational program (i.e., 8 months) was similar to that of this study. The results showed that the level of social self-efficacy significantly increased for the experimental group following participation in the program (medium effect size: Cohen's *d* = 0.54). Similar teaching methods were used for that educational program: “modeling [observational learning through modeling (i.e., watching others do a certain task)], rehearsing (practicing by repetition so as to improve performance; for instance, practicing an action, a play, a conversation, etc.), and verbal rewarding (e.g., verbal praise, positive feedback, realistic encouragement)” (Malinauskas et al., 2018, p. 167). Notably, this allows us to conclude that there is a difference in effectiveness between implementing an educational program in physical education classes and implementing it in the school sports (extracurricular) context.

Elsewhere, a study by Gorucu (2016) investigated the effects of physical education lessons planned in accordance with a cooperative learning approach designed to improve problem-solving skills in secondary school students and reported a large effect size for self-control (Cohen's *d* = 2.14). The study similarly used a model-based practice; however, it differed in the duration of the intervention (only 10 weeks). Meanwhile, the results of a study of 7th-grade students by Goudas et al. (2006) indicated that an experimental group partaking in lifeskills program reported greater personal goal-setting skills compared with the baseline assessment (medium effect size: Cohen's *d* = 0.51) but higher nonsignificant levels of positive-thinking skills (i.e., optimism) compared with the baseline assessment. The life skills program took place over a 1-month period (two sessions per week for 4 weeks) and was based on the school-based intervention designed by Danish et al. (1992) to teach life skills (social skills for life). The study similarly used a model-based practice, also similarly, the intervention included group learning (cooperative learning), post-activity discussion, and written worksheets, with 15-min sessions but different in terms of intervention duration.

Meanwhile, a study by Goudas and Magotsiou (2009) examined the effect of a cooperative physical education program on the social skills of students and their attitudes toward group work, with the results indicating significant increases in the social skills scores of the experimental group following participation in the program for all of the investigated social skills: cooperating skills, empathy, and quick-temperedness (large effect sizes ranging from $\eta_{\text{p}}^{2}$ = 0.26 to $\eta_{\text{p}}^{2}=$ 0.52). The study similarly selected cooperative learning activities (e.g., groups of four, feedback, reciprocal teaching, and alternative roles) but differed in terms of the duration of the intervention (only 5 weeks) and the duration of sessions (45 min).

In conclusion, various studies have found consistent evidence for the positive impacts on student social-emotional competencies and general behavior derived from the social-emotional educational programs in physical education classes. Our finding that youth school students demonstrate more developed social-emotional skills in physical education classes following the intervention could be explained by the Dyson and Casey (2012) model-based practice of cooperative learning, wherein social and affective learning outcomes are combined to develop social-emotional skills as recognized elements of a comprehensive educational program. The results of this study could also be explained by the positive youth development approach, which indicates how skill-building activities, such as possibilities for improving physical, social, and psychological skills in physical education contexts, can prepare students to be competent and healthy (Ebbeck and Gibbons, 1998; Weiss, 2011).

For both the cooperative learning approach and the positive youth development approach, the concept of autonomy-supporting teacher behavior is relevant; this indicates that teacher-student relationships are cooperative, and students feel that behavior is self-determined rather than controlled (Weiss and Wiese-Bjornstal, 2009; Weiss, 2011). The results of a similar study (Papacharisis et al., 2005) show that when social-emotional skills training is properly integrated into physical education classes, trained social-emotional skills are not taught at the expense of motor skills training. These results might be explained by the participatory-learning methods applied, which included listening to instructions about the relevant skills, observing the skills in practice (modeling), practicing the skills in selected situations in a cooperative learning context, and receiving feedback about the individual performance of the skills. Thus, the learning of social-emotional skills was facilitated by the practice of motor skills (Papacharisis et al., 2005).

### Contributions and Implications

First, there is a considerable need to more deeply understand the particularities of training the social-emotional skills of youth school students through physical education classes. Youth school students experienced varying types of risks, hence, the individual discrepancies in their need for social-emotional skills training. Better understanding the social-emotional skills of youth school students in the context of physical education classes can provide insights that can help those students to transform their weaknesses into strengths and overcome the challenges they face, thus further improving their social-emotional skills. Second, this study has demonstrated that a Social-Emotional Skills Training Program can be successfully implemented during physical education classes not only in mainstream secondary schools but also in youth schools.

In general, this study provides important cues for investigators wanting to better understand the importance of social-emotional skills training in youth schools. It has demonstrated that implementing a Social-Emotional Skills Training Program in physical education classes is a powerful factor that can help to enhance the social-emotional skills of youth school students.

### Limitations and Future Research

One of the limitations of this study was that it only considered students of high school age (15–18); further study is needed to analyze the peculiarities of middle- and primary-school-age students in terms of developing social skills in physical education classes. It would also be useful to compare the data among students from different age groups. The self-reported measures used represent another limitation of this study. Further research could also explore the behavioral measures of the use of social-emotional skills of the students in settings other than physical education.

Another potential limitation is that we assigned intact classes to the experimental and the control replication conditions. However, this, in general, corresponds to the likely real situation: the program will usually be implemented within regular classes. In addition, the results showed that the experimental and control groups did not differ before the beginning of the program.

Finally, the lack of retention and transfer measures of social-emotional skills could represent a limitation. Future studies could employ follow-up measures to demonstrate retention.

## Data Availability Statement

The raw data supporting the conclusions of this article will be made available by the authors, without undue reservation.

## Ethics Statement

The studies involving human participants were reviewed and approved by the Ethical Committee of the Lithuanian Sports University. The participants provided their written informed consent to participate in this study. Written informed consent to participate in this study was provided by the participants' legal guardian/next of kin.

## Author Contributions

RM: conceptualization and methodology. VM: data analysis. VM and RM: data collection, writing, original draft preparation, review and editing and visualization and supervision. Both authors contributed to the article and approved the submitted version.

## Conflict of Interest

The authors declare that the research was conducted in the absence of any commercial or financial relationships that could be construed as a potential conflict of interest.

## Publisher's Note

All claims expressed in this article are solely those of the authors and do not necessarily represent those of their affiliated organizations, or those of the publisher, the editors and the reviewers. Any product that may be evaluated in this article, or claim that may be made by its manufacturer, is not guaranteed or endorsed by the publisher.
